# Interleukin-6 Induces S100A9 Expression in Colonic Epithelial Cells through STAT3 Activation in Experimental Ulcerative Colitis

**DOI:** 10.1371/journal.pone.0038801

**Published:** 2012-09-04

**Authors:** Min Jeoung Lee, Jin-Ku Lee, Ji Won Choi, Chang-Seok Lee, Ji Hyun Sim, Chung-Hyun Cho, Kwang-Ho Lee, Ik-Hyun Cho, Myung-Hee Chung, Hang-Rae Kim, Sang-Kyu Ye

**Affiliations:** 1 Department of Pharmacology, Seoul National University College of Medicine, Seoul, Republic of Korea; 2 Ischemic/Hypoxic Disease Institute, Seoul National University College of Medicine, Seoul, Republic of Korea; 3 Department of Anatomy, Seoul National University College of Medicine, Seoul, Republic of Korea; 4 Department of Biotechnology, College of Biomedical & Health Science, Konkuk University, Chungju, Republic of Korea; 5 Department of Anatomy, College of Oriental Medicine, Kyung Hee University, Seoul, Republic of Korea; 6 Institute of Oriental Medicine, Kyung Hee University, Seoul, Republic of Korea; University of Chicago, United States of America

## Abstract

**Background:**

Intestinal epithelium is essential for maintaining normal intestinal homeostasis; its breakdown leads to chronic inflammatory pathologies, such as inflammatory bowel diseases (IBDs). Although high concentrations of S100A9 protein and interleukin-6 (IL-6) are found in patients with IBD, the expression mechanism of S100A9 in colonic epithelial cells (CECs) remains elusive. We investigated the role of IL-6 in S100A9 expression in CECs using a colitis model.

**Methods:**

IL-6 and S100A9 expression, signal transducer and activator of transcription 3 (STAT3) phosphorylation, and infiltration of immune cells were analyzed in mice with dextran sulfate sodium (DSS)-induced colitis. The effects of soluble gp130-Fc protein (sgp130Fc) and S100A9 small interfering (si) RNA (si-S100A9) on DSS-induced colitis were evaluated. The molecular mechanism of S100A9 expression was investigated in an IL-6-treated Caco-2 cell line using chromatin immunoprecipitation assays.

**Results:**

IL-6 concentrations increased significantly in the colon tissues of DSS-treated mice. sgp130Fc or si-S100A9 administration to DSS-treated mice reduced granulocyte infiltration in CECs and induced the down-regulation of S100A9 and colitis disease activity. Treatment with STAT3 inhibitors upon IL-6 stimulation in the Caco-2 cell line demonstrated that IL-6 mediated S100A9 expression through STAT3 activation. Moreover, we found that phospho-STAT3 binds directly to the *S100A9* promoter. S100A9 may recruit immune cells into inflamed colon tissues.

**Conclusions:**

Elevated S100A9 expression in CECs mediated by an IL-6/STAT3 signaling cascade may play an important role in the development of colitis.

## Introduction

Crohn's disease (CD) and ulcerative colitis (UC), which develop as the result of chronic inflammatory reactions in the gastrointestinal tract and are defined collectively as inflammatory bowel disease (IBD), are among the most common autoimmune diseases worldwide [1]–[4]. IBD results from the unregulated response of the mucosal immune system in the gut [5]. It is involved in autoimmune diseases and increases the risk of developing colorectal cancer, one of the most common fatal malignancies [6]. Despite recent advances in our understanding of IBD, important questions about the molecular mechanisms of its immunopathology remain unanswered.

Immune cells, which infiltrate the inflamed guts of patients with IBD, produce various cytokines and chemokines that trigger inflammatory responses [5]. Among the cytokines, interleukin-6 (IL-6) has a positive correlation with the disease activities of IBD, and its production returns to normal levels when gut inflammation becomes inactive [7]–[11]. Of important, IL-6 production was also increased in DSS-induced colitis [11]–[14].

IL-6 plays an important role in enhancing T-cell survival and apoptosis resistance in the lamina propria at the inflamed site [13], [15]. It is also involved in the immune deviation of regulatory T cells toward inflammatory cells (e.g., Th17) [16] and promotes the survival of intestinal epithelial cells [14], [17]. In general, IL-6 binds to soluble or membrane-bound IL-6 receptors (e.g., sIL-6Rα or IL-6Rα), resulting in the activation of janus kinase 2 (JAK2) and the downstream effectors signal transducer and activator of transcription 3 (STAT3) and phosphatidyl inositol 3′ kinase [11], [18]. In particular, genome-wide association studies have found a significant association between polymorphisms in the *STAT3* region and clinical phenotypes of CD and UC, as well as those of multiple sclerosis (MS) [19].

S100A8 (also called myeloid-related protein 8 [MRP8]) and S100A9 (MRP14) are members of the S100 family of calcium-binding proteins and exist mainly as a S100A8/S100A9 heterodimer (i.e., calprotectin) in the extracellular milieu [20]–[22]. They are expressed constitutively in granulocytes, monocytes, and activated macrophages [21]–[27], as well as in epithelial cells under inflammatory conditions [28], [29]. Of important, it has been known that STAT3 regulate the expression of S100A9 in breast cancer cells and myeloid-derived suppressor cells in cancer [30], [31].

The S100A8/S100A9 heterodimer as well as S100A9 induce neutrophil adhesion to fibrinogen by activating the β2 integrin Mac-1 and adhesion molecules (e.g., VCAM-1 and ICAM-1), as well as proinflammatory chemokines [32]–[34]. In addition, this complex functions as an endogenous activator of Toll-like receptor 4 (TLR4), promoting lethal, endotoxin-induced shock [35]. In vascular inflammation, S100A8/S100A9 characteristically damages endothelial integrity and prompts caspase-dependent and -independent cell death [34], [36], [37]. Due to their functions in monocyte activation and leukocyte recruitment, S100A8/S100A9 have been considered hallmarks of many pathologic conditions characterized by chronic inflammation and autoimmunity, such as rheumatoid arthritis, systemic lupus erythematosus, MS, and IBD [29], [38]–[40].

Apart from their known functions under inflammatory conditions, little has been proven about the expression mechanism of S100A9 in intestinal epithelial cells (IECs), which are important in intestinal homeostasis [2], [3]. Because IECs are able to express IL-6Rα on the basal surface, and the ligation of IL-6Rαactivates nuclear factor kappa B (NF-κB) [41], [42], we investigated whether IL-6, which is abundantly expressed in the inflamed colon, modulates the expression of S100A9 in colonic epithelial cells (CECs) using an experimental colitis model. We generated a mouse model of experimental colitis induced by dextran sulfate sodium (DSS) and showed that IL-6 triggered S100A9 production in CECs, which was mediated through STAT3 activation. In addition, we suggest that the increased expression of S100A9 might give rise to the recruitment of immune cells into the colonic epithelial area in this model, resulting in the progression of inflammation.

## Results

### IL-6 is Up-Regulated in the Colon Tissue in DSS-Induced Colitis

We established DSS-induced colitis shown a loss of approximately 20% body weight on day 11(Fig. S1A, left). Notably, the DSS-treated mice scored up to 13 points at the end of the cycle and their DAI scores increased gradually during the course of DSS treatment (Fig. S1A, right). We histologically examined the colon tissues of normal mice and those exposed to DSS for 6 and 12 days. Both groups of DSS-exposed mice showed prominent changes in colon tissues, with mucosal ulceration and degeneration, a decreased number of goblet cells, inflammatory cellular infiltration, and submucosal edema compared to normal mice (Fig. S1B). Histological changes were more severe on day 12, with mucosal ulceration and degeneration as well as inflammatory cellular infiltration into the mucosa and submucosa, indicating that the murine colitis model was well established in our system.

**Figure 1:**
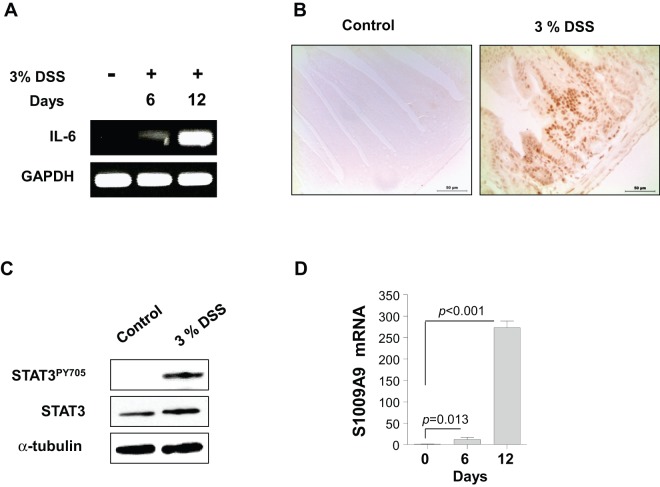
Increased IL-6 expression, activation of STAT3 in colon tissue, and S100A9 in isolated colonic epithelial cells (CECs), from a mouse model of dextran sulfate sodium (DSS)-induced colitis. (**A**) In control and DSS-treated mice at 6 or 12 days after DSS administration, colon tissues were homogenized using a tissue homogenizer. *IL-6* and *GAPDH* mRNA levels in each group of colonic tissue samples was analyzed by conventional reverse-transcription polymerase chain reaction assays. Data are representative of three independent experiments. (**B**) Activation of STAT3 (STAT3^PY705^) in colon sections on day 6 from mice treated with 0% or 3% DSS was determined by immunohistochemistry. Scale bars = 100 µm. Data are representative of four independent experiments. (**C**) CECs from mice treated with 0% or 3% DSS were purified on day 6. Expressions of STAT3^PY705^, total STAT3, and alpha-tubulin were analyzed by immunoblotting. Data are representative of four independent experiments. (**D**) CECs from mice treated with 3% DSS for 0, 6, or 12 days were prepared, and *S100A9* mRNA expression was analyzed by quantitative reverse-transcription polymerase chain reaction (qRT-PCR). The qRT-PCR data were analyzed by comparative C_t_ quantification. Data are presented as means ± standard deviations of values from six mice per group. *P* values were obtained using the two-tailed Student's *t-*test.

To characterize the expression of the effector cytokine IL-6 during DSS-induced colitis, the mRNA expression of IL-6 was evaluated by conventional PCR. We found that IL-6 concentrations increased significantly in the colon tissue of DSS-treated mice (Fig. 1A), although the type of cells contributing to this IL-6 production remained unclear.

### STAT3 Activation and S100A9 Expression in the Colonic Epithelial Cells in DSS-Induced Colitis

To investigate whether STAT3 is activated in CECs, where IL-6 was increased, from DSS-treated mice, we measured STAT3^PY705^ by immunofluorescence and immunoblotting. STAT3 was highly activated in the CEC regions of colon tissues after DSS exposure for 6 days, whereas it was not expressed in control mice (Fig. 1B). To further confirm the activation of STAT3, we isolated CECs from control or DSS-treated mice. The purity of the isolated CECs was confirmed that they express E-cadherin and villin, but not CD45, common leukocyte antigen (Fig S2 A–B). Indeed, STAT3 activation was markedly elevated in the CECs from mice with DSS-induced colitis (Fig. 1C). Since the secretion of S100A9 was correlated with STAT3 [30], [31], we further investigated the expression levels of S100A9 in the CECs. The mRNA expression of S100A9 was strongly elevated in CECs from DSS-treated mice, with the highest expression observed after the longest DSS exposure (Fig. 1D). These data indicate that STAT3 activation may be related to the expression of S100A9 in CECs during DSS-induced colitis.

### IL-6 Blockade or STAT3 Knockdown Suppresses S100A9 Expression in CECs from DSS-Treated Mice

**Figure 2:**
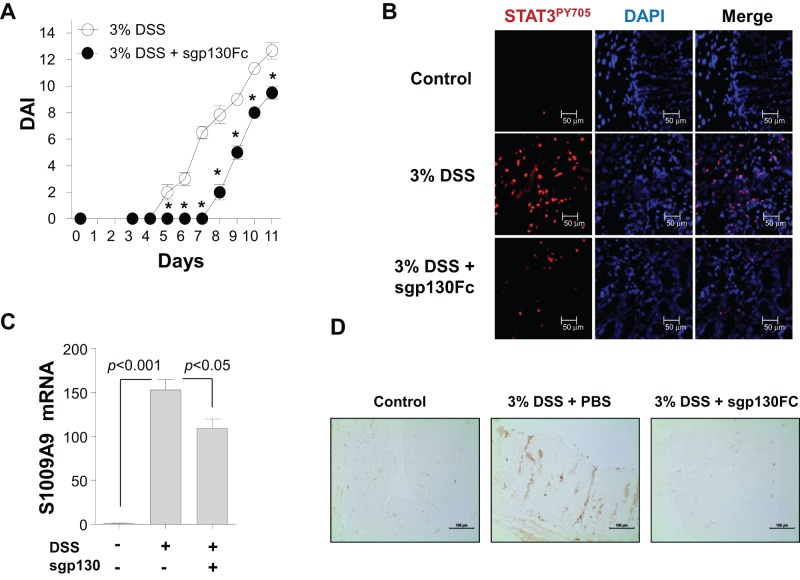
Effect of soluble gp130-Fc (sgp130Fc) injection on the expression of S100A9 in colonic epithelial cells (CECs) from mice with dextran sulfate sodium (DSS)-induced colitis. (**A**) Changes in the disease activity indices (DAI) of mice treated with DSS + phosphate-buffered saline (PBS) or DSS + sgp130Fc were assessed daily. Data are presented as means ± standard deviations of values from six mice per group. *p<0.01 vs. control mice (two-tailed Student's *t-*test). (**B**) Colon sections from mice in each group were examined for STAT3PY705 (red) levels by immunofluorescence on day 6 of DSS treatment. Data are representative of three independent experiments. DAPI, 4′,6-diamidino-2-phenylindole. Scale bars = 50 µm. (**C**) CECs from mice treated with or without 3% DSS for 8 days and a subsequent sgp130Fc or PBS injection were prepared, and *S100A9* mRNA expression was analyzed by quantitative reverse-transcription polymerase chain reaction (qRT-PCR). Data are presented as means ± standard deviations of values from six mice per group. *P* values were obtained using the two-tailed Student's *t-*test. (**D**) Expression of S100A9 in colon sections from mice treated with 0% or 3% DSS for 10 days was determined by immunohistochemistry. Scale bars = 100 µm. Data are representative of four independent experiments.

Based on these findings, the possibility that IL-6 acts as a regulator of S100A9 expression through STAT3 activation in CECs was examined using an IL-6 blockade method. In brief, IL-6 was abrogated by the intraperitoneal injection of 0.5 mg/kg sgp130Fc into a group of mice after 2 days of 3% DSS treatment, as described previously [43]. This method was used because signaling in response to IL-6 involves binding of the cytokine to its receptor (IL-6Rα) and subsequent homodimerization of the signal transducer gp130. Disease severity was significantly reduced in the sgp130Fc-injected group compared to the group receiving DSS alone (Fig. 2A). Then we investigated whether STAT3 phosphorylation could be inhibited by sgp130Fc treatment using immunofluorescence staining. While the expression of STAT3^PY705^ was highly up-regulated in the colon tissues of the 3% DSS-treated group, it was notably repressed in the group of mice treated with 3% DSS and a subsequent injection of sgp130Fc (Fig. 2B). Remarkably, a significant reduction in the expression of *S100A9* mRNA (Fig. 2C) and protein (Fig. 2D) was observed in CECs from the 3% DSS + sgp130Fc group compared to mice receiving DSS alone, and was associated with STAT3^PY705^ expression (Fig. 2B).

**Figure 3:**
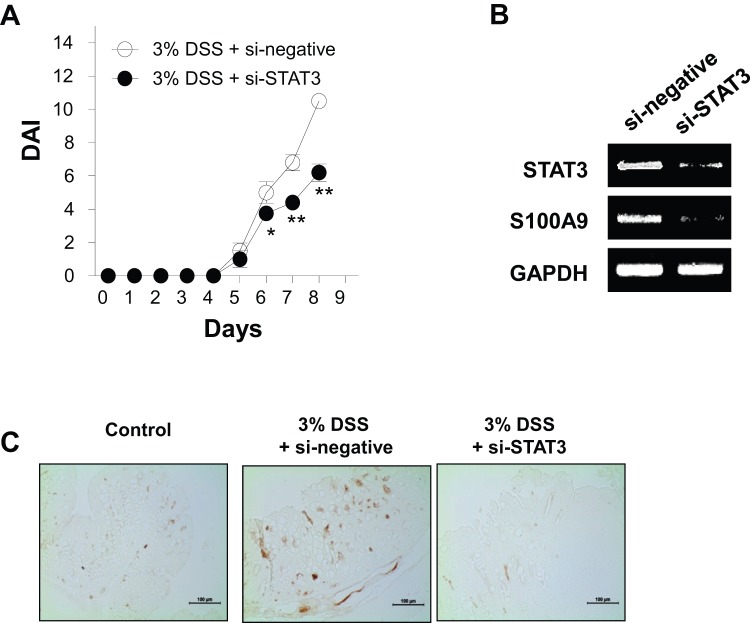
Effect of small interfering STAT3 chitosan nanoparticle (si-STAT3/CH-NP) injection on the expression of S100A9 in colonic epithelial cells (CECs). (**A**) si-negative/CH-NPs or si-STAT3/CH-NPs were injected intravenously twice on days 2 and 3 into 3% DSS-treated mice. Changes in the disease activity indices (DAI) of mice injected with si-negative/CH-NPs or si-STAT3/CH-NPs were assessed daily. Data are presented as means ± standard deviations of values from six mice per group. *p<0.01, **p<0.001 vs. control mice (two-tailed Student's *t-*test). (**B**) On day 8 of DSS exposure, CECs from each group of mice were purified and mRNA levels of *STAT3*, *S100A9*, and *GAPDH* were analyzed by conventional RT-PCR. Data are representative of three independent experiments. (**C**) Expression of S100A9 in colon sections from mice treated with si-negative/CH-NPs or si-STAT3/CH-NPs at 8 days was determined by immunohistochemistry. Scale bars = 100 µm. Data are representative of four independent experiments.

Next, to provide direct evidence that the increased S100A9 expression by IL-6 was mediated through STAT3 activation, we introduced a delivery system of si-STAT3 using CH-NPs [44]. si-negative/CH-NPs or si-STAT3/CH-NPs were injected intravenously twice on days 2 and 3 into the 3% DSS-treated group. Then we also investigated whether STAT3 knockdown could affect disease development using si-STAT injection. Disease activities were significantly decreased on day 6 of DSS exposure in the STAT3 suppression group compared to the negative si-RNA-treated mice (Fig. 3A). The expression levels of *S100A9* mRNA (Fig. 3B) were notably suppressed in the group of mice treated with si-STAT3/CH-NPs, resulting that STAT3 regulated the expression level of S100A9 in the CECs in DSS-induced colitis (Fig. 3C).

### IL-6/STAT3 Signaling Cascades Induce S100A9 Expression in Caco-2 Cells *In Vitro*

**Figure 4:**
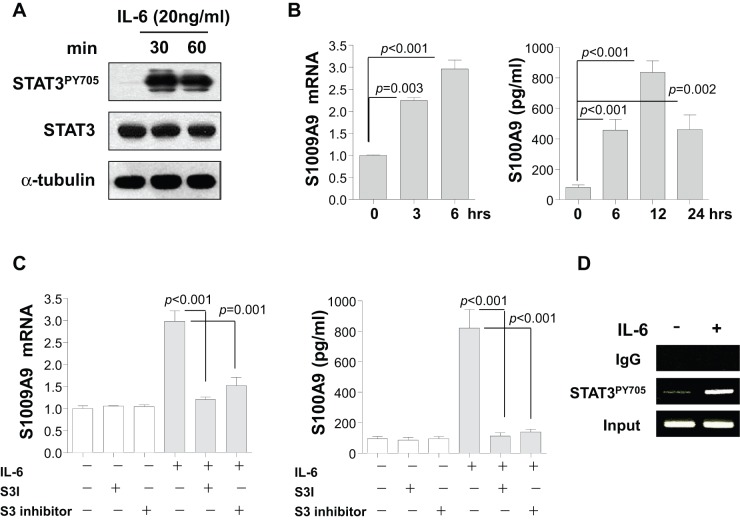
The role of the interleukin-6 (IL-6)/STAT3 axis in the expression of S100A9 in the Caco-2 cell line. (**A**) Caco-2 cells were stimulated with IL-6 (20 ng/ml) for 0, 30, or 60 min, and the cell lysates were analyzed by immunoblotting for STAT3^PY705^ and total STAT3. Data are representative of three independent experiments. (**B**) The expression levels of *S100A9* in Caco-2 cells stimulated with IL-6 (20 ng/ml) for 0, 3, or 6 h were analyzed by quantitative reverse-transcription polymerase chain reaction (qRT-PCR, left panel). The qRT-PCR data were analyzed by comparative C_t_ quantification. S100A9 levels in the culture supernatants of Caco-2 stimulated with IL-6 for 0, 6, 12, or 24 h were determined by ELISA (right panel). Data are presented as means ± standard deviations of values from each group. (**C**) Up-regulation of IL-6-mediated S100A9 expression by STAT3 activation in Caco-2 cells. To measure the expression level of S100A9 in Caco-2 cells, the cells were pretreated with or without one of inhibitors for 3 h and then incubated in the presence or absence of IL-6 (20 ng/ml) for 6 h:S3I (20 µM); or STAT3 inhibitor peptide (5 µM). The transcriptional levels of S100A9 were analyzed by qRT-PCR (left panel) and secreted S100A9 were determined by ELISA (right panel). Data are presented as mean ± standard deviations of values from each group. *P* values were obtained using the two-tailed Student's *t-*test (**B, C**). (**D**) Caco-2 cells were stimulated with or without IL-6 (20 ng/ml) for 24 h. The cell lysates were prepared and sonicated (30 s on/1 min off for three cycles) to form a sheared, cross-linked chromatin. After incubation with anti-STAT3^PY705^ or rabbit immunoglobulin G (IgG), the eluted DNA was amplified and analyzed by conventional polymerase chain reaction assay. Data are representative of four independent experiments.

To further confirm whether IL-6 directly triggers S100A9 expression through STAT3 activation, *in vitro* experiments were performed using a human IEC line (Caco-2). Sustained STAT3 activation was observed until 60 min (Fig. 4A) and S100A9 expression was significantly increased at mRNA levels after IL-6 stimulation for 3 or 6 h (Fig. 4B). S100A9 was released in the cell culture supernatant up to 24 h after stimulation of these cells with IL-6 (Fig. 4B). Thus, IL-6 can be postulated to mediate phosphorylated STAT3 and up-regulate S100A9 expression in Caco-2 cells.

IL-6-mediated S100A9 expression was further investigated to determine whether it is dependent on STAT3 activation using various agents that inhibit STAT3 signaling cascades. S3I and the STAT3 inhibitor, which inhibit phosphorylation by targeting a tyrosine residue or by dimerizing STAT3, respectively were incubated for 3 h prior to IL-6 stimulation. After 6 h, the expression of S100A9 at both mRNA and protein levels was measured. The expression of S100A9, which increased in response to IL-6 stimulation, was returned to resting levels by the two types of STAT3 inhibitors (Fig. 4C). These findings indicate that S100A9 expression by IL-6 in Caco-2 cells was directly mediated via the STAT3 signaling pathway.

To investigate whether STAT3 regulates the transcription of S100A9 upon IL-6 stimulation through direct binding to its promoter region, we performed a ChIP assay. Increased binding of STAT3^PY705^ to the *S100A9* promoter in response to IL-6 was observed (Fig. 4D). Taken together, these data suggest that STAT3 activated in the presence of IL-6 interacts directly with the *S100A9* promoter, resulting in the production of S100A9.

### Leukocytes Infiltration, Presumably Mediated by S100A9, in the Colonic Epithelium in DSS-Induced Colitis

**Figure 5:**
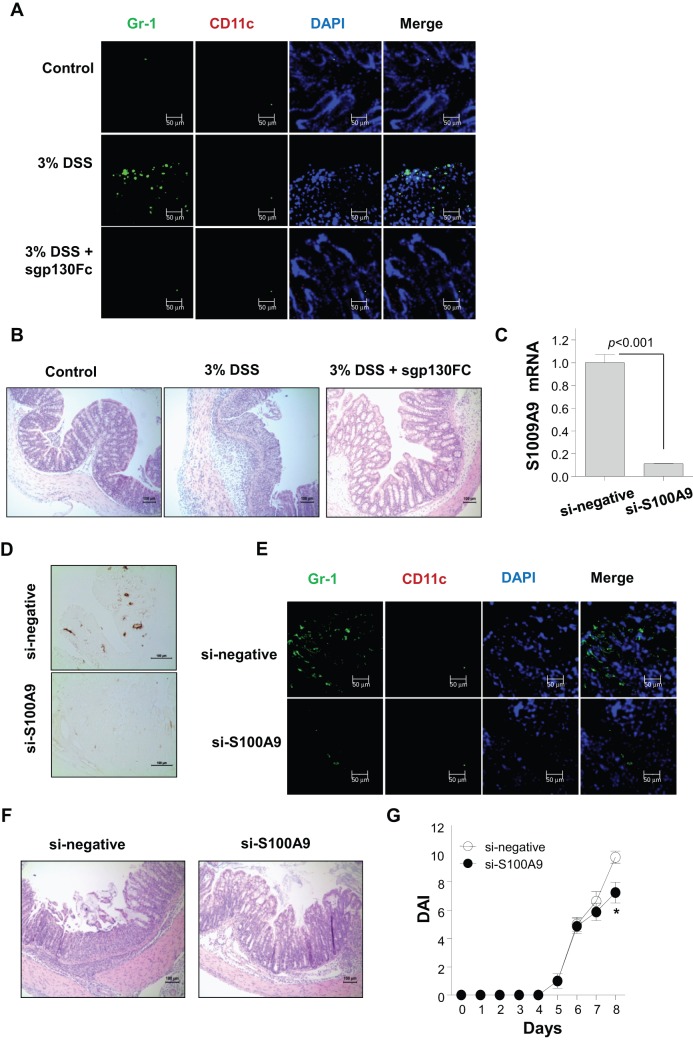
Gr-1^+^ cells infiltration in the colonic epithelial area of mice with dextran sulfate sodium (DSS)-induced colitis. (A) Colon sections from control mice and those treated with 3% DSS + phosphate-buffered saline (PBS) or 3% DSS + soluble gp130-Fc (sgp130Fc) were examined for Gr-1 (green), CD11c (red), and 4′,6-diamidino-2-phenylindole (DAPI; blue) expression by immunofluorescence on day 10 of DSS treatment. Data are representative of three independent experiments. Scale bars = 50 µm. (**B**) Colon histology was examined in control and DSS-treated mice at 10 days after DSS or DSS + sgp130FC administration by hematoxylin and eosin staining of paraffin-embedded sections. Scale bars = 100 µm. Data are representative of three independent experiments. (**C–D**) Small interfering-negative chitosan nanoparticles (si-negative/CH-NPs) or si-S100A9/CH-NPs were injected intravenously twice on days 2 and 3 into 3% DSS-treated mice. On day 10 of DSS-exposure, expression of S100A9 in colon sections from mice was determined by qRT-PCR (C) and immunohistochemistry (D). qRT-PCR data are presented as mean ± standard deviations of values from each group. *P* values were obtained using the two-tailed Student's *t-*test Scale bars = 100 µm. Immunohistochemistry data are representative of four independent experiments. (**E**) The colon sections of DSS-exposed mice receiving si-negative/CH-NP or si-S100A9/CH-NP injections were examined for Gr-1 (green), CD11c (red), and DAPI (blue) expression by immunofluorescence on day 10 of DSS treatment. Data are representative of three independent experiments. Scale bars = 50 µm. (**F**) Colon histology was examined in control and DSS-treated mice at 10 days after si-negative/CH-NP or si-S100A9/CH-NP injections by hematoxylin and eosin staining of paraffin-embedded sections. Scale bars = 100 µm. Data are representative of three independent experiments. (**G**) Changes in the disease activity indices (DAI) of DSS-exposed mice receiving si-negative/CH-NP or si-S100A9/CH-NP injections were assessed daily. Data are presented as mean ± standard deviations of values from six mice per group. *p<0.01 vs. control (two-tailed Student's *t-*test).

Lastly, we explored the effects of S100A9 protein in CECs on DSS-induced colitis. Because the up-regulation of S100A9 is associated exclusively with the recruitment of leukocytes [21], [22], [38], we investigated whether IL-6-mediated S100A9 expression in CECs is associated with the recruitment of immune cell in the colonic epithelial area in DSS-treated mice using immunofluorescence staining for Gr-1 and CD11c. In fact, human recombinant S100A9 were evaluated *in vitro* in chemotaxis assy for human promyelocytic leukemia (HL-60) cell line at concentrations varying from 32 to 1,024 ng/ml. Cell migration was induced directional migration, depending on concentration of S100A9 (Fig. S4). We noticed a significant infiltration of Gr-1^+^ cells in the epithelial lining, but found no expansion of CD11c^+^ cells in DSS-induced colitis (Fig. 5A, E). Importantly, the infiltration of leukocytes in the colonic epithelium (Fig. 5A) was markedly suppressed when IL-6 was blocked by sgp130Fc injection (Fig. 5C). In order to confirm the possibility whether inflammatory chemokine is involved in recruitment of immune cells at inflammatory site, we measured CXCL10 (IP-10) expression. The expression of CXCL10 mRNA was mildly decreased but not significant statistically (Fig S3), suggesting that leukocytes infiltration is secondary to S100A9 expression not chemokine. Moreover, tissue damage in sgp130Fc injected colitis mice was restored to the level of normal mice with alleviated mucosal ulceration and degeneration, a decreased number of goblet cells, inflammatory cellular infiltration, and submucosal edema (Fig. 5B).

To further confirm whether these findings result from S100A9 expression in CECs from mice with DSS-induced colitis, we suppressed S100A9 expression by si-S100A9/CH-NP injection. The expression of S100A9 was markedly reduced by treatment with si-S100A9/CH-NPs (Fig. 5D). In particular, disease activities were significantly decreased on day 8 of DSS exposure in the S100A9 suppression group compared to the negative si-RNA-treated mice (Fig. 5G). The infiltration of GR-1^+^ cells and histopathology of colon tissue were consistently and notably reduced when S100A9 expression was down-regulated (Fig. 5E–F), suggesting that the recruitment of immune cells in the colonic epithelium results from IL-6-induced S100A9 expression in CECs in DSS-induced colitis.

## Discussion

We demonstrated a novel mechanism in which IL-6 increases S100A9 levels via STAT3 activation in CECs in an experimental murine model of DSS-induced colitis. S100A9 expressed in the CECs may contribute to the disease progression of active colitis by recruiting leukocytes within the colonic epithelia, presumably resulting in the breakdown of the epithelial lining and intestinal homeostasis. Our finding that non-immune cells, including mucosal epithelial cells, trigger immune responses through the release of damage-associated signals, such as S100A9, expands our understanding of the pathogenesis of UC in humans.

Although S100A9 is highly up-regulated in patients with UC, and is considered a fecal marker of gastrointestinal inflammation, previous studies have not determined whether CECs express S100A9 in colonic inflammation [22], [45]. A growing body of evidence indicates that ECs help to maintain homeostasis in the gut by acting as physiological barriers against pathogenic bacteria or by generating anti-inflammatory signals. In contrast, our results suggest that ECs can also trigger inflammatory responses by secreting S100A9 in mice with colitis (Fig. 5E–G). Once the epithelial barrier is disrupted, ECs actively secrete damage-associated signals (i.e., S100A9) to prevent potentially pathogenic microorganisms from entering the systemic circulation, which can lead to severe infections, such as peritonitis or sepsis. S100A9, which has been identified as a danger-associated molecular pattern, is recognized by TLR4 in both dendritic cells (DCs) and ECs [21], [22], [46]. The S100A8/S100A9 heterodimer is also an active component that induces a complex signaling cascade comprising the translocation of myeloid differentiation primary response protein 88 and the activation of IL-1 receptor-associated kinase-1, mitogen-activated protein kinases, and the inhibitor of κB kinase by interacting with TLR4-myeloid differentiation factor 2, promoting lethality during septic shock by elevating tumor necrosis factor-α [35]. In addition, S100A9 triggers the infiltration of leukocytes, including granulocytes, which rapidly engulf invaders, damaged cells, or cellular debris [21], [22], [38], [47]. Neutrophil granules also serve as reservoirs for digestive enzymes before delivery into the phagosome. Among them, azurophilic granule contents possess microbicidal activity and play an important role in tissue destruction during inflammation [47]–[49]. Consequently, the infiltration of granulocytes can facilitate the disintegration of the colonic epithelia and exacerbate colitis-associated symptoms in active UC. Moreover, our results suggest that S100A9 blockades, in combination with appropriate antibiotics, may be considered a treatment strategy for patients with UC.

In the present study, we found increased IL-6 expression in CECs from mice with DSS-induced colitis (Fig. 1A) and demonstrated directly that IL-6 induced S100A9 expression in both CECs from mice with colitis and in a human IEC cell line (Fig. 1D, 4B). The activation of TLR4 leads to the induction of IL-6 through the NF-κB signaling pathway [50]. Although CECs express low levels of TLR4 under normal conditions, the expression of TLR4 and IL-6 is increased in CECs from patients with UC, as well as in leukocytes and ECs [12], [51], suggesting that intestinal ECs may produce IL-6 through TLR4 expression during inflammation [52].

Our results provide supporting evidence for the crucial role of IL-6 in the generation of colonic inflammation, as IL-6 blockade delayed disease onset and attenuated colitis-associated symptoms in DSS-treated mice (Fig. 2A). This finding is consistent with Atreya et al. [13], who demonstrated that the blockade of IL-6 *trans-*signaling suppressed T-cell resistance to apoptosis in intestinal inflammation. Collectively, IL-6 elicits colonic inflammation in various ways, such as by stimulating innate immune organs (i.e., epithelial barrier), inducing the differentiation of adoptive immune cells (i.e., Th17 cells), and enhancing T-cell survival. This evidence provides insight into IL-6 function in multiple steps of UC generation and suggests a rationale for therapeutic approaches using IL-6 blockades, which could be effective treatment modalities for patients with UC.

However, IL-6 has a protective effect on enterocytes and IECs [14], [17], [53]. In the development of colitis-associated cancer (CAC), the IL-6/STAT3 axis acts as an oncogenic signal cascade in CECs by promoting BCL-_XL_ and cyclin D [14]. In addition, it has been known that patients with active UC had significantly more IL6 and p-STAT3-positive epithelial cells than both patients with inactive UC and controls [53]. Notably, mice lacking IL-6 (IL-6 null) or STAT3 in IECs show reduced CAC tumorigenesis but develop more severe DSS-induced colitis, with pronounced colonic ulceration and body weight loss, than do wild-type counterparts [14]. Tebbutt et al. [54] also showed that IL-6 null mice or those harboring the reciprocal mutation ablating STAT1/3 signaling show impaired colonic mucosal wound healing after DSS administration.

There are several possible explanations for the difference between our findings and the results of previous studies of the blockade of IL-6 signaling or STAT3 activation. It could stem from the use of knockout mice, the administration of the soluble receptor blocker (sgp130Fc), or different methods of targeting the S100A9 molecule. In intestinal inflammation, S100A9 is an effector molecule that enhances TLR signaling [35] and recruits granulocytes [21], [22], [38], [47]. Thus, the blockade of this molecule can ameliorate disease severity in DSS-induced colitis (Fig. 5A–C). Thus, our data and previous findings suggest that IL-6 can finely tune the balance between negative and positive signals during DSS-induced colitis by providing protective signals to IECs or by promoting inflammation via interaction with CECs and inflammatory cells.

In conclusion, our results suggest the existence of an immune-triggering mechanism mediated by S100A9 released by CECs that helps to scavenge invading microorganisms when epithelial barriers are impaired. This mechanism may also induce further undesirable destruction in epithelial linings, leading to the exacerbation of colitis symptoms. Further studies should investigate how this mechanism can be controlled without interfering with immune surveillance in CECs. Our findings suggest that drugs inhibiting STAT3 signals may be good candidates for controlling disease activities in patients with UC while avoiding CAC development.

## Materials and Methods

### Cell Culture

A Caco-2 human IEC line was obtained from the Korean Cell Line Bank (Seoul, Republic of Korea). Human embryonic kidney cell line 293T and human colon cancer cell line HCT116 were purchased from the American Type Culture Collection (Manassas, VA, USA). The cells were cultured in appropriate media with 10% heat-inactivated fetal bovine serum (Lonza, Walkersville, MD, USA).

### Reagents and Inhibitors

DSS (molecular weight, 35,000–50,000 Da; United States Biochemical, Cleveland, OH, USA) was used to induce experimental colitis in mice. IL-6 (PeproTech, Rocky Hill, NJ, USA) was used to stimulate Caco-2 cells. The following inhibitors were used to treat Caco-2 cells throughout IL-6 stimulation: S3I and STAT3 inhibitor peptide (Merck) to block phosphorylation by targeting a tyrosine residue and to dimerize STAT3, respectively [55], [56].

To prepare small interfering (si) RNA-incorporated chitosan nanoparticles (CH-NPs), siRNAs for STAT3 and S100A9 were purchased from Bioneer Corporation (Daejeon, Republic of Korea). siRNA-incorporated CH-NPs were prepared according to a previously described method [44] by ionic gelation of anionic tripolyphosphate (TPP; Sigma Chemical, St. Louis, MO, USA) and siRNA with cationic chitosan (Sigma Chemical). The weight ratio of chitosan to TPP was set to 3∶1 to generate particles about 200∶nm in size. The prepared siRNA/CH-NPs (si-STAT3/CH-NPs and si-S100A9/CH-NPs) were injected intravenously twice into mice with DSS-induced colitis.

### Establishment of an Experimental DSS-Induced Colitis Mouse Model

Six-week-old male C57BL/6 mice were purchased from Joongang Laboratory Animal Co. (Seoul, Republic of Korea) and maintained under specific pathogen-free conditions. As described previously [57], colitis was induced with water containing 3% DSS. The disease activity index (DAI) represents the combined scores of weight loss, stool consistency, and bleeding, according to scoring criteria described previously [58], [59]. Body weight loss (scores: 0, none; 1, 1–10%; 2, 11–20%), stool consistency (scores: 0, normal; 1, soft; 2, liquid), hemoccult positivity, and the presence of blood (scores: 0, negative fecal occult blood; 1, positive fecal occult blood; 2, visible rectal bleeding) were assessed daily in each group of mice. In some experiments, mice were injected intraperitoneally with soluble gp130-FC fusion protein (sgp130Fc, 0.5 mg/kg; R&D Systems, Minneapolis, MN, USA) or phosphate-buffered saline (PBS). All surgical and experimental procedures were reviewed and approved by the Institutional Animal Care and Use Committee (IACUC) in College of Medicine, Seoul National University.

### Isolation of Colonic Epithelial Cells

CECs were isolated using a modified version of previously described methods [60], [61]. In brief, colon pieces were incubated in Ca^2+^- and Mg^2+^-free Hanks Balanced Salt Solution (HBSS; Sigma Chemical) containing 2% calf serum at 37°C for 30 min while stirring. After collecting the supernatant, remaining tissue was resuspended in HBSS with 3 mM ethylenediaminetetraacetic acid (EDTA). The tissues were incubated while stirring at 37°C for 30 min. The supernatant was then collected and centrifuged at 300×g for 10 min.

### Histopathology and Immunohistochemistry of Colon Tissue

Large intestine specimens were fixed in 10% neutralized formalin for 24 h, embedded in paraffin, and sectioned at 4 µm. Colon sections were stained with hematoxylin and eosin (H&E) for histopathological analysis, and stained immunohistochemically as described previously to investigate the expression of phospho-STAT3 (STAT3^PY705^) [62]. Briefly, paraffin-embedded slides were hydrated with ethanol and distilled water, followed by antigen retrieval in 10 mM sodium citrate buffer. Sections were incubated with anti-S100A9 (Abcam, Cambridge, MA, USA) and anti-STAT3^PY705^ immunoglobulin G (IgG; Cell Signaling Technology, Danvers, MA, USA) and stained with 3,3-diaminobenzidine substrate. Image acquisition and processing were performed using a Leica microscope and the Leica Application Suite (Leica Microsystems, Buffalo Grove, IL, USA).

### Immunofluorescence of Colon Tissue

To perform immunofluorescence analyses, mouse large intestine specimens were embedded in optimal cutting temperature compound (Sakura Finetek Japan, Tokyo, Japan) and sectioned at 10 µm by cryostats (Leica Microsystems). The sections were incubated with a combination of anti-Gr-1-biotin and purified anti-CD11c antibodies (BD Biosciences) or with rabbit anti-STAT3^PY705^ IgG at 4°C overnight, followed by incubation with appropriate secondary antibodies. Stained sections were mounted in VectaShield 4′, 6-diamidino-2-phenylindole (DAPI) mounting medium (Vector Laboratories, Burlingame, CA, USA) and analyzed under a confocal laser scanning microscope (LSM 510; Carl Zeiss, Gottingen, Germany). Images were taken under a confocal microscope using a 40× objective.

### Immunoblotting

Cells were harvested in a lysis solution (Santa Cruz Biotechnology, Santa Cruz, CA, USA) in the presence of a protease inhibitor cocktail (Roche, Basel, Switzerland) and phosphatase inhibitor (Santa Cruz Biotechnology). After incubation for 30 min on ice, insoluble debris was removed by centrifugation for 15 min at 4°C. Total protein was resolved by sodium dodecyl sulfate polyacrylamide gel electrophoresis and transferred to nitrocellulose membranes (GE Healthcare, Pittsburgh, PA, USA). The membranes were then probed with antibodies against α-tubulin (Thermo Fisher Scientific, Fremont, CA, USA), STAT3 (Cell Signaling Technology), or STAT3^PY705^ and visualized using SuperSignal West Femto Chemiluminescent Substrate (Thermo Fisher Scientific).

### Quantitative and Conventional Reverse-Transcription Polymerase Chain Reaction Assays

Total RNA was isolated from colon tissues and cDNA was synthesized using a QuantiTech Reverse Transcription Kit (QIAGEN, Valencia, CA, USA), then mixed with QuantiFast SYBR Green polymerase chain reaction (PCR) master mix (QIAGEN) and specific primers. Quantitative reverse-transcription PCR (qRT-PCR) was performed with an Applied Biosystems 7300 Real-Time PCR System (Life Technologies, Carlsbad, CA, USA) and analyzed by comparative C_t_ quantification [63]. The conventional PCR products were generated by *AccuPower* PCR Premix (Bioneer). The specific primers for genes used in this study were obtained from QIAGEN.

### Enzyme-linked Immunosorbent Assay (ELISA)

Cell culture supernatants were analyzed for S100A9 levels using an S100A9 ELISA kit (Cyclex Co., Ltd, Nagano, Japan) according to the manufacturer's instructions.

### Chromatin Immunoprecipitation

Chromatin immunoprecipitation (ChIP) assays were performed using a Chromatin Immunoprecipitation Assay Kit (Millipore, Billerica, MA, USA) according to the manufacturer's instructions. Briefly, Caco-2 cells were fixed with formaldehyde for 10 min at room temperature. After sonication (30 s on/1 min off for three cycles), the fragmented soluble chromatin was immunoprecipitated with anti-STAT3^PY705^ IgG or rabbit IgG. Cross-links were reversed by incubating at 65°C. Precipitated DNA was amplified by conventional PCR using specific primers covering the canonical STAT3 binding motif within the *S100A9* promoter: forward, 5′-ACACATCTTGCCCACCAGGAGGCTA-3′; reverse, 5′-AAATCCTGCTCCCAGCAAGGGTTCC-3′.

### Statistical Analysis

Data are presented as means ± standard deviations (SDs). *P* values <0.05 were considered statistically significant. Two-tailed Student's *t-*tests were conducted using the GraphPad Prism software (ver. 5.01; GraphPad Software, La Jolla, CA, USA).

## Supporting Information

Figure S1 **Changes in interleukin-6 (IL-6) expression in the colons of an experimental ulcerative colitis mouse model established by dextran sulfate sodium (DSS) treatment.** (**A**) The difference (%) between baseline and subsequent body weight was measured daily for 11 days (left). Changes in clinical symptoms are represented by the disease activity index (DAI; right). Data are presented as means ± standard deviations of values from six mice per group. *p<0.01, **p<0.001 vs. control mice (two-tailed Student's *t-*test). PBS, phosphate-buffered saline. (**B**) Colon histology was examined in control and DSS-treated mice at 6 or 12 days after DSS administration by hematoxylin and eosin (H&E) staining of paraffin-embedded sections. Scale bars = 100 µm. Data are representative of three independent experiments. (**C**) In each of three groups, colon tissues were homogenized using a tissue homogenizer. *IL-6* and *GAPDH* mRNA levels in each group of colonic tissue samples was analyzed by conventional reverse-transcription polymerase chain reaction assays. Data are representative of three independent experiments.
(EPS)

Figure S2 **The purity of the isolated colonic epithelial cells (CECs) from a mouse model of dextran sulfate sodium (DSS)-induced colitis.** (**A**) Epithelial cells were isolated from colon tissue specimens from DSS-treated or control mice, and the purity of the isolated CECs was confirmed by staining with fluorescein isothiocyanate-labeled antibodies against E-cadherin (BD Biosciences, San Diego, CA, USA) by flow cytometry. Numbers indicate the percentage of positive cells in the histogram. Data are representative of three independent experiments. (**B**) *CD45* and *villin* mRNA expression was analyzed by conventional reverse-transcription polymerase chain reaction (RT-PCR) for isolated CECs and intraepithelial lymphocyte (IEL), which is control cell expressing CD45.
(EPS)

Figure S3 **Effect of soluble gp130-Fc (sgp130Fc) injection on the expression of CXCL10 in colonic epithelial cells (CECs) from mice with dextran sulfate sodium (DSS)-induced colitis.** CECs from mice treated with or without 3% DSS for 8 days and a subsequent sgp130Fc or PBS injection were prepared, and *CXCL10* mRNA expression was analyzed by quantitative reverse-transcription polymerase chain reaction (qRT-PCR). Data are presented as means ± standard deviations of values from six mice per group. *P* values were obtained using the two-tailed Student's *t-*test.
(EPS)

Figure S4 **S100A9 is potent chemotactic factors for human promyelocytic leukemia (HL-60)** ***in vitro*** **.** Chemotaxis assays were performed using CytoSelect Cell migratory Assay Kit (Cell Biolabs, San Diego, CA, USA). Media (control) or increasing concentration of S100A9 (Cyclex Co. Nagano, Japan) were added to the lower chamber. HL-60 cells were added to the upper chamber (5×10^3^ cells) and allowed to migrate through the membrane (5 µm pore size) for 6 h. Migrated cells were stained with trypan blue and counted with hemocytometer. Results are the mean % of migrated cells. Data are presented as means ± standard deviations of values from triplicate experiments. *P* values were obtained using the two-tailed Student's *t-*test.
(EPS)

## References

[1] Podolsky DK (1991). Inflammatory bowel disease (1).. N Engl J Med.

[2] Maloy KJ, Powrie F (2011). Intestinal homeostasis and its breakdown in inflammatory bowel disease.. Nature.

[3] Kaser A, Zeissig S, Blumberg RS (2010). Inflammatory bowel disease.. Annu Rev Immunol.

[4] Garud S, Peppercorn MA (2009). Ulcerative colitis: current treatment strategies and future prospects.. Therap Adv Gastroenterol.

[5] Baumgart DC, Carding SR (2007). Inflammatory bowel disease: cause and immunobiology.. Lancet.

[6] Eaden JA, Abrams KR, Mayberry JF (2001). The risk of colorectal cancer in ulcerative colitis: a meta-analysis.. Gut.

[7] Suzuki Y, Saito H, Kasanuki J, Kishimoto T, Tamura Y (1990). Significant increase of interleukin 6 production in blood mononuclear leukocytes obtained from patients with active inflammatory bowel disease.. Life Sci.

[8] Mudter J, Neurath MF (2007). Il-6 signaling in inflammatory bowel disease: pathophysiological role and clinical relevance.. Inflamm Bowel Dis.

[9] Reinecker HC, Steffen M, Witthoeft T, Pflueger I, Schreiber S (1993). Enhanced secretion of tumour necrosis factor-alpha, IL-6, and IL-1 beta by isolated lamina propria mononuclear cells from patients with ulcerative colitis and Crohn's disease.. Clin Exp Immunol.

[10] Umehara Y, Kudo M, Nakaoka R, Kawasaki T, Shiomi M (2006). Serum proinflammatory cytokines and adhesion molecules in ulcerative colitis.. Hepatogastroenterology.

[11] Matsumoto S, Hara T, Mitsuyama K, Yamamoto M, Tsuruta O (2010). Essential roles of IL-6 trans-signaling in colonic epithelial cells, induced by the IL-6/soluble-IL-6 receptor derived from lamina propria macrophages, on the development of colitis-associated premalignant cancer in a murine model.. J Immunol.

[12] Cammarota R, Bertolini V, Pennesi G, Bucci EO, Gottardi O (2010). The tumor microenvironment of colorectal cancer: stromal TLR-4 expression as a potential prognostic marker.. J Transl Med.

[13] Atreya R, Mudter J, Finotto S, Mullberg J, Jostock T (2000). Blockade of interleukin 6 trans signaling suppresses T-cell resistance against apoptosis in chronic intestinal inflammation: evidence in crohn disease and experimental colitis in vivo.. Nat Med.

[14] Grivennikov S, Karin E, Terzic J, Mucida D, Yu GY (2009). IL-6 and Stat3 are required for survival of intestinal epithelial cells and development of colitis-associated cancer.. Cancer Cell.

[15] Neurath MF, Finotto S, Fuss I, Boirivant M, Galle PR (2001). Regulation of T-cell apoptosis in inflammatory bowel disease: to die or not to die, that is the mucosal question.. Trends Immunol.

[16] Bettelli E, Carrier Y, Gao W, Korn T, Strom TB (2006). Reciprocal developmental pathways for the generation of pathogenic effector TH17 and regulatory T cells.. Nature.

[17] Jin X, Zimmers TA, Zhang Z, Pierce RH, Koniaris LG (2010). Interleukin-6 is an important in vivo inhibitor of intestinal epithelial cell death in mice.. Gut.

[18] Kishimoto T (2005). Interleukin-6: from basic science to medicine–40 years in immunology.. Annu Rev Immunol.

[19] Cenit MC, Alcina A, Marquez A, Mendoza JL, Diaz-Rubio M (2010). STAT3 locus in inflammatory bowel disease and multiple sclerosis susceptibility.. Genes Immun.

[20] Odink K, Cerletti N, Bruggen J, Clerc RG, Tarcsay L (1987). Two calcium-binding proteins in infiltrate macrophages of rheumatoid arthritis.. Nature.

[21] Foell D, Wittkowski H, Vogl T, Roth J (2007). S100 proteins expressed in phagocytes: a novel group of damage-associated molecular pattern molecules.. J Leukoc Biol.

[22] Foell D, Wittkowski H, Roth J (2009). Monitoring disease activity by stool analyses: from occult blood to molecular markers of intestinal inflammation and damage.. Gut.

[23] Thorey IS, Roth J, Regenbogen J, Halle JP, Bittner M (2001). The Ca2+-binding proteins S100A8 and S100A9 are encoded by novel injury-regulated genes.. J Biol Chem.

[24] Rammes A, Roth J, Goebeler M, Klempt M, Hartmann M (1997). Myeloid-related protein (MRP) 8 and MRP14, calcium-binding proteins of the S100 family, are secreted by activated monocytes via a novel, tubulin-dependent pathway.. J Biol Chem.

[25] Katz AB, Taichman LB (1999). A partial catalog of proteins secreted by epidermal keratinocytes in culture.. J Invest Dermatol.

[26] Wilkinson MM, Busuttil A, Hayward C, Brock DJ, Dorin JR (1988). Expression pattern of two related cystic fibrosis-associated calcium-binding proteins in normal and abnormal tissues.. J Cell Sci 91 (Pt.

[27] Roth J, Burwinkel F, van den Bos C, Goebeler M, Vollmer E (1993). MRP8 and MRP14, S-100-like proteins associated with myeloid differentiation, are translocated to plasma membrane and intermediate filaments in a calcium-dependent manner.. Blood.

[28] Frosch M, Metze D, Foell D, Vogl T, Sorg C (2005). Early activation of cutaneous vessels and epithelial cells is characteristic of acute systemic onset juvenile idiopathic arthritis.. Exp Dermatol.

[29] Lugering N, Stoll R, Kucharzik T, Schmid KW, Rohlmann G (1995). Immunohistochemical distribution and serum levels of the Ca(2+)-binding proteins MRP8, MRP14 and their heterodimeric form MRP8/14 in Crohn's disease.. Digestion.

[30] Li C, Zhang F, Lin M, Liu J (2004). Induction of S100A9 gene expression by cytokine oncostatin M in breast cancer cells through the STAT3 signaling cascade.. Breast Cancer Res Treat.

[31] Cheng P, Corzo CA, Luetteke N, Yu B, Nagaraj S (2008). Inhibition of dendritic cell differentiation and accumulation of myeloid-derived suppressor cells in cancer is regulated by S100A9 protein.. J Exp Med.

[32] Guignard F, Mauel J, Markert M (1995). Identification and characterization of a novel human neutrophil protein related to the S100 family.. Biochem J 309 (Pt.

[33] Newton RA, Hogg N (1998). The human S100 protein MRP-14 is a novel activator of the beta 2 integrin Mac-1 on neutrophils.. J Immunol.

[34] Viemann D, Barczyk K, Vogl T, Fischer U, Sunderkotter C (2007). MRP8/MRP14 impairs endothelial integrity and induces a caspase-dependent and -independent cell death program.. Blood.

[35] Vogl T, Tenbrock K, Ludwig S, Leukert N, Ehrhardt C (2007). Mrp8 and Mrp14 are endogenous activators of Toll-like receptor 4, promoting lethal, endotoxin-induced shock.. Nat Med.

[36] Viemann D, Strey A, Janning A, Jurk K, Klimmek K (2005). Myeloid-related proteins 8 and 14 induce a specific inflammatory response in human microvascular endothelial cells.. Blood.

[37] Croce K, Gao H, Wang Y, Mooroka T, Sakuma M (2009). Myeloid-related protein-8/14 is critical for the biological response to vascular injury.. Circulation.

[38] Ryckman C, Vandal K, Rouleau P, Talbot M, Tessier PA (2003). Proinflammatory activities of S100: proteins S100A8, S100A9, and S100A8/A9 induce neutrophil chemotaxis and adhesion.. J Immunol.

[39] Bogumil T, Rieckmann P, Kubuschok B, Felgenhauer K, Bruck W (1998). Serum levels of macrophage-derived protein MRP-8/14 are elevated in active multiple sclerosis.. Neurosci Lett.

[40] Kuruto R, Nozawa R, Takeishi K, Arai K, Yokota T (1990). Myeloid calcium binding proteins: expression in the differentiated HL-60 cells and detection in sera of patients with connective tissue diseases.. J Biochem.

[41] Wang L, Walia B, Evans J, Gewirtz AT, Merlin D (2003). IL-6 induces NF-kappa B activation in the intestinal epithelia.. J Immunol.

[42] Becker C, Fantini MC, Schramm C, Lehr HA, Wirtz S (2004). TGF-beta suppresses tumor progression in colon cancer by inhibition of IL-6 trans-signaling.. Immunity.

[43] Jostock T, Mullberg J, Ozbek S, Atreya R, Blinn G (2001). Soluble gp130 is the natural inhibitor of soluble interleukin-6 receptor transsignaling responses.. Eur J Biochem.

[44] Lu C, Han HD, Mangala LS, Ali-Fehmi R, Newton CS (2010). Regulation of tumor angiogenesis by EZH2.. Cancer Cell.

[45] Fagerhol MK (2000). Calprotectin, a faecal marker of organic gastrointestinal abnormality.. Lancet.

[46] Ehrchen JM, Sunderkotter C, Foell D, Vogl T, Roth J (2009). The endogenous Toll-like receptor 4 agonist S100A8/S100A9 (calprotectin) as innate amplifier of infection, autoimmunity, and cancer.. J Leukoc Biol.

[47] Dale DC, Boxer L, Liles WC (2008). The phagocytes: neutrophils and monocytes.. Blood.

[48] Elsbach P, Weiss J (1998). Role of the bactericidal/permeability-increasing protein in host defence.. Curr Opin Immunol.

[49] Ganz T, Selsted ME, Szklarek D, Harwig SS, Daher K (1985). Defensins. Natural peptide antibiotics of human neutrophils.. J Clin Invest.

[50] Rose-John S, Mitsuyama K, Matsumoto S, Thaiss WM, Scheller J (2009). Interleukin-6 trans-signaling and colonic cancer associated with inflammatory bowel disease.. Curr Pharm Des.

[51] Soliman A, Michelsen KS, Karahashi H, Lu J, Meng FJ (2010). Platelet-activating factor induces TLR4 expression in intestinal epithelial cells: implication for the pathogenesis of necrotizing enterocolitis.. PLoS One.

[52] Abreu MT, Thomas LS, Arnold ET, Lukasek K, Michelsen KS (2003). TLR signaling at the intestinal epithelial interface.. J Endotoxin Res.

[53] Li Y, de Haar C, Chen M, Deuring J, Gerrits MM (2010). Disease-related expression of the IL6/STAT3/SOCS3 signalling pathway in ulcerative colitis and ulcerative colitis-related carcinogenesis.. Gut.

[54] Tebbutt NC, Giraud AS, Inglese M, Jenkins B, Waring P (2002). Reciprocal regulation of gastrointestinal homeostasis by SHP2 and STAT-mediated trefoil gene activation in gp130 mutant mice.. Nat Med.

[55] Miyamoto N, Sugita K, Goi K, Inukai T, Lijima K (2001). The JAK2 inhibitor AG490 predominantly abrogates the growth of human B-precursor leukemic cells with 11q23 translocation or Philadelphia chromosome.. Leukemia.

[56] Turkson J, Ryan D, Kim JS, Zhang Y, Chen Z (2001). Phosphotyrosyl peptides block Stat3-mediated DNA binding activity, gene regulation, and cell transformation.. J Biol Chem.

[57] Okayasu I, Hatakeyama S, Yamada M, Ohkusa T, Inagaki Y (1990). A novel method in the induction of reliable experimental acute and chronic ulcerative colitis in mice.. Gastroenterology.

[58] Cooper HS, Murthy SN, Shah RS, Sedergran DJ (1993). Clinicopathologic study of dextran sulfate sodium experimental murine colitis.. Lab Invest.

[59] Suzuki K, Sun X, Nagata M, Kawase T, Yamaguchi H (2011). Analysis of intestinal fibrosis in chronic colitis in mice induced by dextran sulfate sodium.. Pathol Int.

[60] Montufar-Solis D, Klein JR (2006). An improved method for isolating intraepithelial lymphocytes (IELs) from the murine small intestine with consistently high purity.. J Immunol Methods.

[61] Panja A, Blumberg RS, Balk SP, Mayer L (1993). CD1d is involved in T cell-intestinal epithelial cell interactions.. J Exp Med.

[62] Oskouian B, Sooriyakumaran P, Borowsky AD, Crans A, Dillard-Telm L (2006). Sphingosine-1-phosphate lyase potentiates apoptosis via p53- and p38-dependent pathways and is down-regulated in colon cancer.. Proc Natl Acad Sci U S A.

[63] Popivanova BK, Kitamura K, Wu Y, Kondo T, Kagaya T (2008). Blocking TNF-alpha in mice reduces colorectal carcinogenesis associated with chronic colitis.. J Clin Invest.

